# Three-Dimensional Reservoir-Based Dielectrophoresis (rDEP) for Enhanced Particle Enrichment

**DOI:** 10.3390/mi9030123

**Published:** 2018-03-10

**Authors:** Akshay Kale, Saurin Patel, Xiangchun Xuan

**Affiliations:** 1 Department of Mechanical Engineering, Clemson University, Clemson, SC 29634-0921, USA; saurinp@g.clemson.edu; 2 Department of Chemical Engineering & Biotechnology, University of Cambridge, Cambridge CB3 0AS, UK

**Keywords:** dielectrophoresis, electrokinetics, particle focusing, particle trapping, microfluidics

## Abstract

Selective enrichment of target species is crucial for a wide variety of engineering systems for improved performance of subsequent processes. Dielectrophoresis (DEP) is a powerful electrokinetic method that can be used to focus, trap, concentrate, and separate a variety of species in a label-free manner. The commonly employed methods for DEP suffer from limitations such as electrode fouling and high susceptibility to Joule heating effects. Recently, our group has demonstrated DEP-based manipulations of particles and cells using a novel method of reservoir-based dielectrophoresis (rDEP) which exploits the naturally produced electric field gradients at the reservoir-microchannel junction. Although this method reasonably addresses the limitations mentioned above while maintaining a high simplicity of fabrication, all of our demonstrations so far have used a two-dimensional rDEP, which limits the performance of the devices. This work aims to improve their performance further by making the DEP three-dimensional. Through detailed experimental and numerical analysis, we demonstrate a six-fold increase in the enrichment performance of latex beads and a significant reduction in the power consumption for the new devices, which would allow a more reliable integration of the same into micro-total analysis systems.

## 1. Introduction

Selective enrichment of desired species is a highly beneficial step for enhancing the performance of a technology requiring these species as an input for subsequent processing. For instance, a sensor designed to detect the presence of bacteria would perform better if the target pathogens could be enriched before passing through the sensing system [1]. Likewise, a system designed to culture viable stem cells for their use in regenerative medicine would benefit greatly from their pre-concentrated population [2]. Similar to the aforementioned examples, pre-concentration is useful for a wide variety of applications in the domains of healthcare, biotechnology, food and water quality control [3,4,5,6], etc. The development of microfluidic technology has provided a major breakthrough for the efforts towards this direction by scaling the conventional laboratory processes down to a portable chip. Not only does this greatly reduce the reagent consumption and response timescales compared to the macroscopic laboratory steps [7,8], but it also facilitates a massive parallelization of several steps [9], which facilitates an easy integration of the pre-concentration step with any downstream processing. 

This process of “On-chip” pre-concentration is achieved by several methods such as magnetic [10,11], acoustic [12,13], hydrodynamic [14,15], and optical [16,17], all of which bring about a focusing of the species to a confined volume inside the microfluidic channel, or trapping at specific locations inside the fluid flow. Electric field driven methods [18] are highly preferred over the above methods for achieving pre-concentration because of the spatially uniform electroosmotic fluid flow they produce inside microfluidic channels (which minimizes dispersion issues) and their capacity for an easier integration with downstream processes [19]. Among these, dielectrophoresis (DEP) based methods, which exploit forces induced on polarizable species in non-uniform electric fields [20,21], are particularly useful owing to their label-free nature [22,23]. The dielectrophoretic force **F**_DEP_ acting on a polarizable sphere of diameter d in an electric field **E** is written as:

(1) FDEP=π4εfd3Re(εs∗−εf∗εs∗+2εf∗)〈∇E2〉

where $\varepsilon_{f}$ is the electric permittivity of the liquid, and the quantity in the bracket is the Clausius-Mossotti factor, f_CM_, that depends upon the complex permittivity values of the liquid and the sphere, i.e., $\varepsilon_{f}{}^{*}$ and $\varepsilon_{s}{}^{*}$, respectively. $\langle \nabla E^{2}\rangle$ denotes the gradient of the square of the root mean square (RMS) electric field, and is non-zero for both the direct current (DC) and alternating current (AC) electric fields. 

Conventionally, DEP is generated through different mechanisms of inducing electric field gradients, such as fabricating metallic microelectrodes [24,25,26,27,28,29,30] (also called electrode-based DEP or eDEP), introducing curvatures in microfluidic channels [31,32,33] (also called curvature induced DEP or C-iDEP), or fabricating non-uniform microchannel cross sections within electrical insulators [34,35,36,37,38,39,40,41,42,43,44,45,46] (also called insulator-based DEP or iDEP). The eDEP- and C-iDEP-based methods respectively suffer from limitations of electrode fouling in the region of interest and inability to trap species [47]. The iDEP-based methods are capable of mitigating both of these limitations. However, the voltages required in iDEP devices are very high, which increases the Joule heat generation [48,49,50,51,52]. Additionally, the regions of localized heating, i.e., constrictions, are fabricated within the small volume of the fluidic channel, so that the heat dissipation is a challenging issue. To overcome these limitations, our group developed a novel idea of exploiting the naturally huge size mismatch present at the junction of the microfluidic channel and the fluid reservoir for DEP [53]. The electric field gradients produced locally due to this can be used to induce a so-called reservoir-based DEP or rDEP [54]. The manipulation of target species in these devices occurs near the large volume of the reservoir which acts as a heat sink to reduce the risk of Joule heating effects [55]. In addition, the channel clogging issues [56] are significantly mitigated due to the large volume of the reservoir. In our previous works, we have extensively demonstrated the application of rDEP for separating target cells and particles from the undesirable species and thereby enriching them [53,54,55,56,57]. However, all the above works use the commonly employed technique of punching the reservoir within a larger circular guide fabricated along with the master mold of the fluidic channel, thereby making the reservoir-microchannel junction uniform in depth. This makes the electric field gradients two-dimensional, which puts a limitation on the strength of the so called 2DrDEP obtained.

This work attempts to enhance the performance of 2DrDEP further by making the electric field gradients three-dimensional. This is achieved by designing the reservoir directly at the junction, so that the large height of the reservoir relative to the channel introduces a size mismatch in the third direction as well. Through a combined experimental and numerical study of latex beads as a proof of concept, we report a significant enhancement in the ability of this 3DrDEP to enrich the target species. 

## 2. Experiment

**Figure 1:**
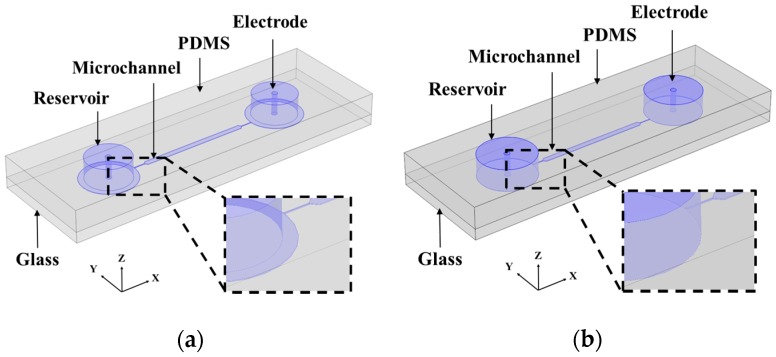
Conceptual schematic of the two-dimensional reservoir-based dielectrophoresis (2DrDEP) and three-dimensional reservoir-based dielectrophoresis (3DrDEP) microfluidic devices. The inset images represent a zoomed view of the reservoir microchannel junction for the device. (**a**) 2DrDEP device fabricated by punching a fluid reservoir within the circular guides patterned as extensions to the microchannel during photolithography. This makes the patterned reservoir-microchannel junction two-dimensional; (**b**) 3DrDEP device fabricated by punching a fluid reservoir directly on the circular guides patterned as extensions to the microchannel during photolithography. This makes the patterned reservoir-microchannel junction three-dimensional.

Figure 1 represents a schematic of the microfluidic device used for the present work. It consists of a fluidic channel having a straight central section that is 1 cm long and 500 µm wide. At each end of this section is a straight constriction that is 1 mm long and 50 µm wide. The central section transitions into the constrictions over a taper angle of 45°. At the other ends of the constrictions, the channel consists of circular extensions with a diameter of 5 mm for establishing a connection with the fluid reservoirs. The channel has a uniform thickness of 40 µm over its entire length. The microfluidic chip was fabricated using the standard soft lithography method as described elsewhere [58,59]. Two types of devices were fabricated. For introducing a 2DrDEP phenomenon, cylindrical reservoirs with a diameter of 4 mm were punched over the circular extensions of the fluidic channel at an offset from their junction with the constrictions. (See inset of Figure 1a for the reservoir structure). For introducing a 3DrDEP phenomenon, cylindrical reservoirs with a diameter of 5 mm were punched carefully enough to coincide with the circular extensions (See inset of Figure 1b for the reservoir structure). While the former device generates a 2D size mismatch at the constriction, the latter adds a third dimension to the mismatch and hence enhances the electric field gradients further.

A 78:22 mixture by volume of 1 mM phosphate buffer and glycerol (Fisher Scientific, Pittsburgh, PA, USA) was prepared and used as the working fluid for the experiments. The conductivity of the fluid was measured as 156 µS/cm (Accumet AP85, Fisher Scientific). Latex beads with a diameter of 5 µm diameter (Sigma-Aldrich, St. Louis, MO, USA) were introduced inside the working fluid at a concentration of 10^6^–10^7^ particles per mL. The mixing of phosphate buffer and glycerol in the above proportion ensured that the beads were neutrally buoyant inside the fluid to avoid sedimentation with time. Prior to the experiments, the channels were primed with the working fluid alone for mitigating the hydrophobicity of their polydimethylsiloxane (PDMS) walls. This fluid was then replaced immediately by the fluid sample containing the suspension of beads. The flow due to a hydrostatic pressure difference was eliminated by carefully balancing the liquid levels in the reservoirs. 

Platinum electrodes of a 0.5 mm diameter were inserted into the reservoirs in order to establish an electrical contact of the fluid with a high-voltage amplifier (609E-6, Trek, Medina, NY, USA) coupled to a function generator (33220A, Agilent Technologies, Santa Clara, CA, USA). DC voltages biased with AC voltages were applied across the electrodes during the experiments. The AC voltage component was responsible for enhancing the DEP phenomenon without altering the electrokinetic flow of the fluid and the beads produced by the DC component. Due to the limited slew rate of our amplifier, the frequency of AC voltages was fixed to 1 kHz in our experiment. However, its variation from 0.5 kHz (lower than this value caused apparent particle oscillations) to 5 kHz (the maximum value we could use at high AC voltages) was not found to affect the particle motion visibly. The particle motion at the reservoir-microchannel junction was recorded with a charge-coupled-device (CCD) camera (DS-Qi 1 Mc, Nikon, Tokyo, Japan) attached to an inverted microscope (Nikon TE2000U, Nikon Instruments, Lewisville, TX, USA), and the videos were transferred to image processing software (NIS-Elements AR 3.22, Nikon, Tokyo, Japan) for post-processing.

## 3. Theory and Simulation

### 3.1. Working Principle

**Figure 2:**
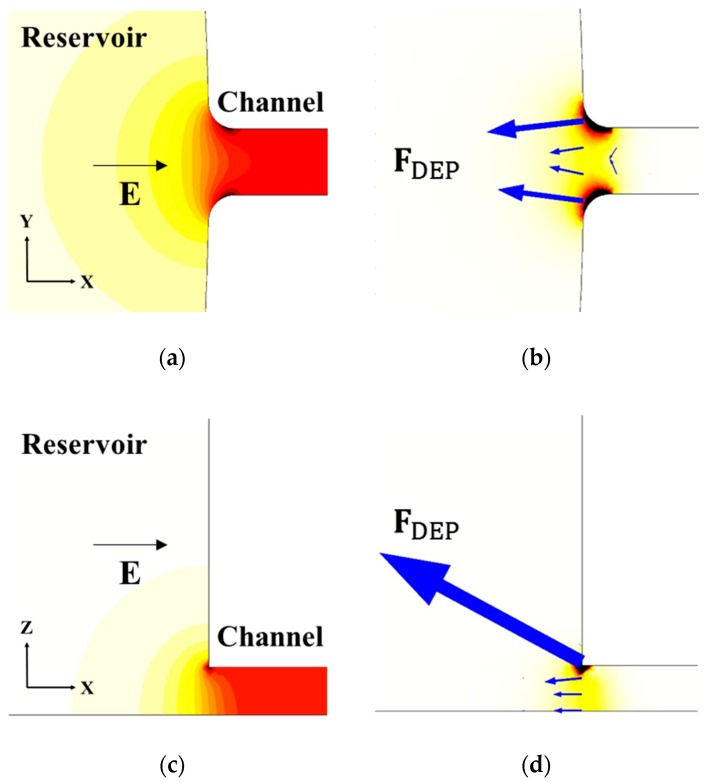
Illustrative mechanism of the proposed three-dimensional reservoir-based dielectrophoresis (3DrDEP) via the views of the reservoir-microchannel junction from different perspectives: (**a**) Top view (X-Y) of the contours of electric field; (**b**) Top view (X-Y) of the rDEP force vectors acting on latex beads; (**c**) Side view (X-Z) of the contours of electric field; (**d**) Side view (X-Z) of the rDEP force vectors acting on latex beads. The contour maps in (**b**) and (**d**) each show the magnitude of the resultant DEP force.

As mentioned previously, a necessary condition for DEP to occur is the presence of non-uniform electric fields. This condition is inherently satisfied due to the electrically insulating nature of PDMS/Glass and the significant size mismatch between the reservoir and the fluidic channel. Hence, as shown in Figure 2, the electric field magnitude **E** inside the fluid gets enhanced around the reservoir-microchannel junction over a sharp gradient represented by the electric field contours (see Figure 2a,c). Any polarizable species moving electrokinetically inside the fluid experiences a dielectrophoretic force in the vicinity of the gradient (see Figure 2b,d). The electrokinetic velocity **U**_EK_ of the species is a function of the electric field at any point, and can be expressed using Equation (2):

(2) $$U_{\mathrm{EK}}={µ}_{\mathrm{EK}}E_{\mathrm{DC}}$$

where $E_{\mathrm{DC}}$ represents the DC component of the electric field, and ${µ}_{\mathrm{EK}}$ represents the electrokinetic mobility of the species, defined as the speed of the species per unit electric field intensity. Due to the linear relation of the electrokinetic motion with **E**, the equilibrium path of the species aligns with the electric field lines. 

The dielectrophoretic force acting on the species is given by Equation (1), and deflects the species from the equilibrium path in a direction determined by the sign of the real part of the Clausius-Mossotti factor, f_CM_. Using the definition of complex permittivities of the species and the fluid, f_CM_ can be expressed as given in Equation (3):

(3) $$f_{\mathrm{CM}}=\left(\frac{\varepsilon_{s}{}^{*}-\varepsilon_{f}{}^{*}}{\varepsilon_{s}{}^{*}+2\varepsilon_{f}{}^{*}}\right)=\left[\frac{\left(\varepsilon_{s}+j\frac{\sigma_{s}}{2\pi f}\right)-\left(\varepsilon_{f}+j\frac{\sigma_{f}}{2\pi f}\right)}{\left(\varepsilon_{s}+j\frac{\sigma_{s}}{2\pi f}\right)+2\left(\varepsilon_{f}+j\frac{\sigma_{f}}{2\pi f}\right)}\right]$$

where $\varepsilon_{s}$ and $\varepsilon_{f}$ are the electric permittivities of the sphere and the fluid, respectively; $\sigma_{s}=4k_{S}/d$ and $\sigma_{f}$ are the electrical conductivities of the sphere and the fluid, respectively; f is the frequency of the applied AC voltage; d is the diameter of the sphere; and $k_{S}$ is its surface conductance (~1 nS [47]). Equation (3) is first employed to calculate the real part of f_CM_, which is then used in Equation (1) to compute the DEP force. A species undergoing electrokinetic motion undergoes a continuous acceleration over its dielectrophoretically deflected path. Since the time scale for this acceleration is very small (~1 µs owing to its micro-scale characteristic lengths [47]), the species always appears to move inside the liquid at a terminal velocity **U**_s_, given by Equation (4):

(4) $$U_{S}=U_{\mathrm{EK}}+\frac{F_{\mathrm{DEP}}}{3\pi \eta_{f}d}$$

where $\eta_{f}$ is the dynamic viscosity of the fluid.

For a DC electric field biased with an AC field of frequency of 1 kHz, 5 µm latex beads exhibit a negative DEP [Real (f_CM_) = −0.4625], and are therefore always deflected away from the reservoir-microchannel junction. For 2DrDEP, as extensively demonstrated in our previous works [53,54,55,56,57], the latex beads are deflected towards the center of the fluid domain (see Figure 2b). In this work, however, the three-dimensional size mismatch produced by the reservoir punch in the 3DrDEP device also introduces a depth-wise gradient at the junction (see Figure 2c), causing the beads to deflect towards the bottom of the channel as well (see Figure 2d). As we demonstrate in this work, this additional deflection component significantly enhances the ability of the device to manipulate the beads. 

### 3.2. Numerical Model

To support the experiments, three-dimensional finite element simulations of the devices (see Figure 1) were generated using COMSOL Multiphysics 4.4 (Burlington, MA, USA). Due to the low frequency and electrical conductivity of the liquid, the electrodynamic and the electric field screening effects were neglected in the model, and the electric field **E** inside the liquid was solved by the Electrostatics interface using the Laplace equation for the voltage V ($\nabla^{2}V=0;E=-\nabla V$). It was then used to compute the velocity of particles (Equation (4)) using the particle tracing function. In order to incorporate the DEP force perturbation effects due to the finite size of the beads, the dielectrophoretic motion component (Second term on the RHS of Equation (4) was multiplied by a correction factor, c, for the simulations. The value of the correction factor inversely depends on the extent of the fluid volume occupied by a particle in the vicinity of the non-uniform cross sections of the channel and therefore reduces for larger particle sizes. The correction factor takes a maximum value of 1 when the electric field perturbation caused by the particles is negligible (i.e., for a point particle assumption). The correction factor is determined by a visual inspection of the numerically predicted DEP path-lines of particles for a given device, and then by tuning the factor to a constant value which is able to provide the closest possible agreement with the experiments. This method has been extensively demonstrated to reasonably predict the experimental particle path-lines in our previous DEP-based works [53,54,55,56,57]. The model was solved only for the DC component of the electric field and the dielectrophoretic effects of the AC field were incorporated into the model by defining an RMS AC to DC voltage ratio, *r*. With these considerations and combining Equations (1)–(4), the equation for the particle motion modified for the purpose of simulation may be written as below:

(5) $$U_{S}={µ}_{\mathrm{EK}}E_{\mathrm{DC}}+\frac{c\varepsilon_{f}d^{2}\mathrm{Real}\left(f_{\mathrm{CM}}\right)\nabla E_{\mathrm{DC}}{}^{2}}{12\eta_{f}}\left(1+r^{2}\right)$$

As a boundary condition, all the channel walls were assumed to be electrically insulating. Due to the absence of an electric field inside the platinum electrodes, they were modeled as holes inside the geometry. The electric fields were obtained by applying the Dirichlet boundary conditions for the required DC electric potential drop across the surfaces of these holes.

An experimentally measured electrokinetic mobility value of 10^−8^ m^2^/Vs was incorporated in the model. The measurement was performed by pumping the fluid-particle mixture through a straight microchannel of a uniform cross section using very low DC voltages, and then correlating the measured bead velocity with the electric field inside that channel. A dynamic viscosity of 2 mN·s/m^2^ (Corresponding to a 78:22 mixture of aqueous buffer and glycerol by volume [60]) and a fluid dielectric constant of 80 were also used in the model. A correction factor of 0.7 was found to reasonably predict the experimentally obtained particle pathlines for all the experiments. This value is comparable to those used in our previous studies [53,54,55,56,57]. Note that Joule heating effects were neglected in our model considering the strong cooling effects of the fluid in the reservoir [55]. This was verified by the insignificant increase in electric current during the course of any test.

## 4. Results and Discussion

### 4.1. Comparison of the Focusing Ability of the Two Devices

**Figure 3:**
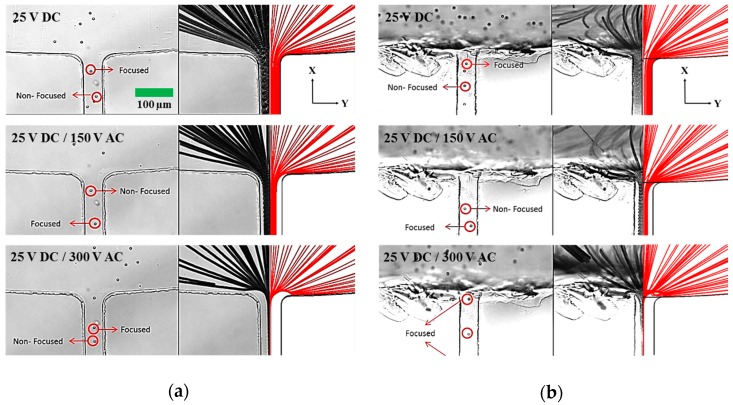
Comparison of the experimentally obtained and numerically predicted path-lines of 5 µm latex beads at the reservoir-microchannel junction of (**a**) 2DrDEP device and (**b**) 3DrDEP device plotted in the top view (X-Y). Three voltage settings are used for comparison, namely 25 V DC, 25 V DC/150 V AC, and 25 V DC/300 V AC. Note that in both devices, the rDEP focusing effect increases with an increase in AC voltage, and the beads get deflected towards the center-line of the channel. Also observe that for all the voltages, some of the focused beads in the 2DrDEP device (**a**) appear blurred in the field of view of the microscope, indicating an absence of a three-dimensional focusing. In contrast, all the beads in the 3DrDEP device (**b**) appear sharply focused at higher voltages, indicating a three-dimensional focusing effect towards the channel center-line and the channel bottom.

The comparison between 2DrDEP and 3DrDEP on 5 µm beads at the reservoir-microchannel junction as a function of applied voltages can be seen in Figure 3. The DC voltage is fixed at 25 V, and two AC voltages of 150 V and 300 V are superimposed over the same. The effects are compared against the situation of a pure DC voltage. A good agreement is observed between the experimentally obtained and numerically predicted particle path-lines for each device. As seen in Figure 3a, the electric field gradients introduced for a pure DC voltage are negligible, and one can observe the beads occupying the entire width of the channel. Recalling the direction of the DEP force on the beads in the X-Y plane (Figure 2b), we term the beads along the center-line of the channel as focused and the remaining ones as non-focused. The effect of superimposing an AC voltage over the DC voltage is immediately evident, and a voltage of 150 V AC is seen to partially focus the beads towards the centre of the channel. Hence, only a fraction of the channel width is now occupied by the beads. However, some non-focused beads can still be observed. Finally, at 300 V AC, the beads are seen to become strongly focused and hence form a thin streak almost coinciding with the center-line of the channel. 

At any voltage, however, it can also be seen from Figure 3a that some of the beads appear blurred while some appear sharp in the field of view, indicating that they lie in different planes along the depth of the channel. This also implies that the 2DrDEP device does not deflect the beads in the depth-wise direction, due to which the focusing is only limited to the X-Y plane (see Figure 2b). This is consistent with the geometry of the reservoir-microchannel junction that does not change its dimension along the depth-wise direction. The aforementioned limitation of the focusing ability of the 2DrDEP device is significantly mitigated by making the reservoir-microchannel junction three-dimensional. Figure 3b represents the experimentally obtained and numerically predicted DEP-induced particle motion for the 3DrDEP device obtained by punching the reservoir directly at the junction. The applied voltages are identical to those in the former device. It can be seen that the modified geometry of the device not only helps retain the X-Y plane focusing of the beads, but also introduces an additional focusing effect in the Z direction towards the channel bottom (Recall Figure 2d). This is evident from the fact that the beads not only flow along the center-line of the channel, but also appear sharp under the field of view of the microscope when it is focused upon the channel bottom.

To quantify the focusing performance of the two devices mentioned above, we define a dimensionless focusing effectiveness χ, as the inverse of the area fraction of the fluid domain occupied by the beads after they are focused at the junction. Thus, we can write:

(6) $$\chi =\frac{Area\;of\;the\;channel\;cross-section}{Area\;occupied\;by\;the\;beads\;in\;the\;channel\;cross-section}=\frac{W_{Ch}\times H_{Ch}}{W_{Bead}\times H_{Bead}}$$

where $W_{Ch}$ and $W_{Bead}$ are the widths of the channel and the focused streak of particles in the X-Y plane, respectively; and $H_{Ch}$ and $H_{Bead}$ are the thickness of the channel and the focused streak of particles in the X-Z plane, respectively. Clearly, χ≥ 1 and a higher value of χ indicates a better focusing capacity of the device.

**Figure 4:**
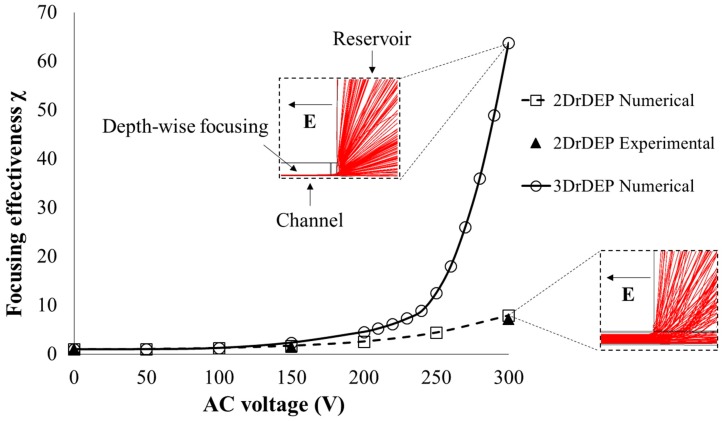
Graphical representation of the variation of the dimensionless focusing effectiveness, χ (defined in Equation (6)), as a function of the applied AC voltage in the 2DrDEP and the 3DrDEP devices. The applied DC voltage is fixed at 25 V, while the AC voltage is varied from 0 V to 300 V. Numerically predicted 5 µm particle path-lines are used to measure the width and depth of the focused particles and the measurements are used in Equation (6) to calculate the effectiveness. Three experimental data points corresponding to the images in Figure 3a, are also shown for comparison with the numerical graphs for 2DrDEP. These experimental data points are obtained by measuring the experimentally generated streak width of the beads in the top view (X-Y), and recalling that the depth of this focused streak is equal to the channel height due to the 2D electric field gradients. The measured streak widths are then substituted in Equation (6) to obtain the focusing effectiveness. The inset images represent the numerically predicted path-lines for 25 V DC/300 V AC plotted in the side view (X-Z). Observe the significant increase in the focusing effectiveness of the 3DrDEP device over the 2DrDEP device because of the strong additional depth-wise component of the electric field gradients produced in the former.

**Figure 5:**
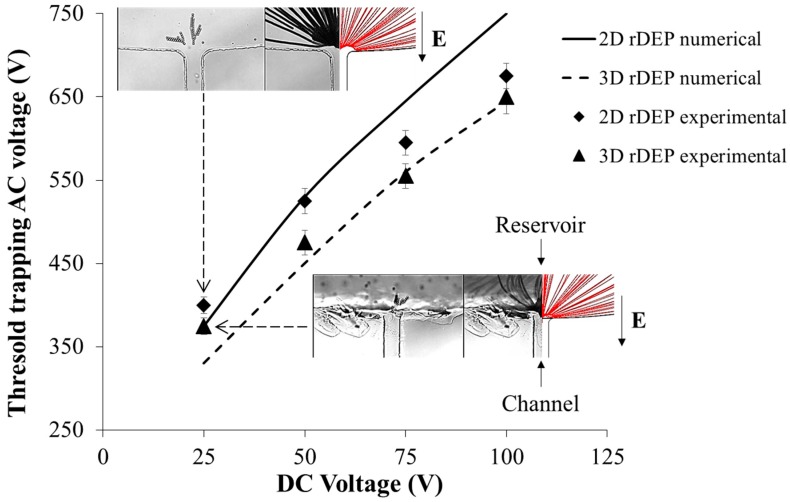
Graphical representation of the variation of the threshold AC voltage required to trap the 5 µm beads as a function of the applied DC voltage in the 2DrDEP and the 3DrDEP devices. The applied DC voltages are varied from 25 V to 100 V in steps of 25 V, while the AC voltage is varied from 0 V until the value where trapping is achieved. The inset images represent the comparison of experimentally obtained and numerically predicted path-lines for an applied DC voltage of 25 V DC plotted in the top view (X-Y). Observe the significant reduction in the AC voltage required for trapping inside the 3DrDEP device over that in the 2DrDEP device. This is because of the strong additional depth-wise component of the electric field gradients produced in the former, which reduces the requirement of applied voltage for producing comparable DEP forces.

Figure 4 compares the numerically predicted focusing effectiveness χ of the 2DrDEP and 3DrDEP devices as a function of the applied electric potential. Having fixed the DC voltage at 25 V, the AC voltage is increased from 0 V (Pure DC case) to 300 V in steps of 50 V. Simulations of particle pathlines (X-Z view) at 25 V DC/300 V AC are shown for both the devices (see inset images) for a visual comparison. The focusing effectiveness values calculated using the experimentally observed particle path-lines at 0 V, 150 V, and 300 V AC inside the 2DrDEP device (Figure 3a) are also shown for comparison with the 2DrDEP numerical data, and both are seen to be in very good agreement with each other. The corresponding experimental data points for the 3DrDEP device could not be obtained (Figure 3b) due to the inability of the microscope to allow observations in the side view (X-Z). We are currently developing a method to visualize the particle motion at the reservoir-microchannel junction in the depth direction by the use of a prism that is placed closely besides the channel. The value of χ at each numerical data point is calculated by measuring the width and depth of the particle streak-line plot generated from the simulation, and then substituting those values in Equation (6). The focused particle widths required for the experimental data points were measured directly from the particle streak images in Figure 3a. Although it was not possible to measure the depths of the streak experimentally, it was safely assumed that the beads always occupied the entire channel height considering the 2D nature of the electric field gradients (Also confirmed by Figure 3a and the inset image in Figure 5). 

As demonstrated previously, the latex beads occupy the entire channel cross-section for a pure DC voltage, so that according to Equation (6), the focusing effectiveness is at its minimum value of unity. As one increases the AC voltage over the DC voltage, the beads begin to experience an rDEP focusing and this causes them to occupy a smaller cross-sectional area than that of the fluid itself. Hence the focusing effectiveness of the devices begins to increase above 1. It can be seen that up to an AC voltage of 200 V, the difference between the focusing effectiveness of the two devices is not significant, indicating that DEP is dominated by the two dimensional X-Y focusing effect. However, above this voltage, the three-dimensional electric field gradients of the 3DrDEP device are able to strongly focus the beads towards the channel bottom, and confine them to a significantly smaller area inside the liquid. Hence the focusing effectiveness of the 3DrDEP device increases rapidly over that of the 2DrDEP device. At 25 V DC/300 V AC, the 3DrDEP device is seen to focus the beads about six times better than the 2DrDEP device, which is indicative of the substantial advantage offered by the method of punching the reservoir directly at the reservoir-microchannel junction. Note that the degree of geometric confinement is essentially identical between our 2DrDEP and 3DrDEP devices, where the only difference lies in the position of the reservoir. 

### 4.2. Comparison of the Trapping Ability of the Two Devices

Figure 5 represents a graphical comparison of the AC voltages required to trap 5 µm beads in the two devices for different values of the DC voltage offset. Four DC voltages are tested, namely, 25 V, 50 V, 75 V, and 100 V, and for each value, the AC voltage is successively increased up to a critical value where the rDEP becomes strong enough to surpass the focusing effect and completely trap the particles at the reservoir-microchannel junction. These critical values are then plotted as a function of the DC voltages for both the devices. It can be seen that the numerically predicted trapping AC voltage curves diverge from each other as one increases the DC voltage. This trend is consistent with the physics of the rDEP mechanism, and can be explained from the DEP force equation (Equation (1)). The electric field gradients for a 3DrDEP device are stronger than those in the 2DrDEP device at any given DC voltage, and hence from Equation (1), a lower AC voltage should be sufficient to produce comparable DEP forces and trap the beads in the former. Likewise, as the DC voltage is increased, it begins to produce its own electric field gradients, and hence the trapping AC voltages required in the 3DrDEP device should become increasingly lower than the corresponding AC voltages in the 2DrDEP device. This should cause the curves to diverge from each other.

Considering the tolerance limits, the experimentally obtained trapping AC voltages are seen to provide a reasonable agreement with the simulation data at lower DC voltages in Figure 5. At higher voltages, however, the 2DrDEP numerical data overpredicts the experimental data slightly. A possible reason for this discrepancy could be the pressure driven back-flows created by pumping the liquid continuously while seeking the trapping AC voltages. A strong electrokinetic flow at higher DC voltages in both devices would induce a pressure driven back-flow of the liquid, which would reduce the apparent electrokinetic velocity of the beads focused at the channel center-line [61]. However, the size mismatch of the 3D reservoir-microchannel junction is much larger compared to that of the 2D junction and hence the hydraulic resistance of the 2DrDEP device would be lower. This would induce a stronger back-flow in the 2D rDEP device compared to that in the 3DrDEP device, so that a lower AC voltage would be needed than expected to trap the beads. However, both the experimental and numerical data predict very well that the 3DrDEP device would consume significantly lower powers for trapping the beads than a 2DrDEP device for a given throughput (controlled by the DC voltage). In addition, the 3DrDEP device is also expected to be less susceptible to the inevitable Joule heating effects which scale with the square of the applied voltage [47,48,49,50,51,52]. This is particularly beneficial while using the devices for biological applications where maintaining the viability of the species is crucial.

It is important to note that the trapping efficiency of 5 µm beads is 100% in both the 2DrDEP and 3DrDEP devices. In fact, once multiple beads get trapped at the reservoir-microchannel junction, they start forming chains and clusters (see the inset images in Figure 5) that can further disturb the local electric field for an increasingly easier trapping [58]. The enriched beads can be released to the microchannel for analysis at any time by simply decreasing the AC voltage (hence reducing the DEP force) or increasing the DC voltage (hence enhancing the electrokinetic flow) if they have reached a sufficient number or need to be cycled to avoid blockage. However, as the particle trapping takes place completely inside the reservoir, whose volume is orders of magnitude larger than that of a microchannel, we believe that the cycling period of our 3DrDEP device should be substantially longer than that of any other microchannel-based trapping methods. The flow throughput of our device was estimated to be around 7 µL/h at 100 V DC due to the relatively slow electrokinetic flow. This value can be readily increased to ~1 mL/h range by the use of multiple parallel microchannels in a multi-layer-stacked device, which has been recently demonstrated for 2DrDEP [57]. The particle throughput of our 3DrDEP device is dependent on the concentration of particles, which essentially has no limit in the trapping process until the reservoir is fully saturated with particles. 

## 5. Concluding Remarks

The present work demonstrates a more effective method to dielectrophoretically enrich polarizable species at the reservoir-microchannel junction. Using latex beads as a proof of concept, we successfully demonstrate the crucial role played by a three-dimensional reservoir-microchannel junction in enhancing the dielectrophoretic pre-concentration potential of the devices. Using the quantification of the focusing effectiveness of the devices, we successfully demonstrate a six-fold increase in the focusing ability of a 3DrDEP microfluidic device over its 2DrDEP counterpart for a given applied voltage. An additional advantage of the 3DrDEP device lies in the fact that it confines the focused beads along the bottom channel wall. This provides promising opportunities for integrating it with other downstream processes, e.g., a detection system [62] along that wall for achieving a highly enhanced detection performance, or a cell culture chamber in which the enriched target species would already be in a sedimented state [63] for facilitating culturing. In addition, the 3DrDEP device is also shown to consume lower electrical powers for dielectrophoretic trapping of the beads for a given throughput/flow rate, which would facilitate its usage over a wider range of operating powers before it would begin exhibiting the undesirable Joule heating effects. At the same time, however, it must be noted that although the proposed method can prolong the heating effects from manifesting in terms of the applied voltages, it cannot completely eliminate them. The method would work very well for DEP applications involving cells suspended in low to moderate conductivity buffers. However, the use of this method for scenarios such as the enrichment of small sized species or enrichment in high conductivity media needs a more general study of the method with the inclusion of Joule heating and electrothermal effects [64,65]. Additionally, it would also be essential to evaluate the enrichment performance of the method in positive DEP, where the channel clogging issues would still be significant. In the near future, we intend to extend the present proof of concept and generalize this technology for enriching any given species and determine input design limitations specific to the same.
